# ROS production and signalling in chloroplasts: cornerstones and evolving concepts

**DOI:** 10.1111/tpj.15856

**Published:** 2022-06-28

**Authors:** Christine H. Foyer, Guy Hanke

**Affiliations:** ^1^ School of Biosciences, College of Life and Environmental Sciences University of Birmingham Edgbaston B15 2TT UK; ^2^ School of Biological and Chemical Sciences Queen Mary University of London Mile End Road London E1 4NS UK

**Keywords:** antioxidants, cytochrome *b*_6_ *f* complex, hydrogen peroxide, peroxiredoxins, photosynthesis, Photosystem II, Photosystem I, redox signalling, singlet oxygen, superoxide, thioredoxins

## Abstract

Reactive oxygen species (ROS) such as singlet oxygen, superoxide (O_2_^●−^) and hydrogen peroxide (H_2_O_2_) are the markers of living cells. Oxygenic photosynthesis produces ROS in abundance, which act as a readout of a functional electron transport system and metabolism. The concept that photosynthetic ROS production is a major driving force in chloroplast to nucleus retrograde signalling is embedded in the literature, as is the role of chloroplasts as environmental sensors. The different complexes and components of the photosynthetic electron transport chain (PETC) regulate O_2_^●−^ production in relation to light energy availability and the redox state of the stromal Cys‐based redox systems. All of the ROS generated in chloroplasts have the potential to act as signals and there are many sulphhydryl‐containing proteins and peptides in chloroplasts that have the potential to act as H_2_O_2_ sensors and function in signal transduction. While ROS may directly move out of the chloroplasts to other cellular compartments, ROS signalling pathways can only be triggered if appropriate ROS‐sensing proteins are present at or near the site of ROS production. Chloroplast antioxidant systems serve either to propagate these signals or to remove excess ROS that cannot effectively be harnessed in signalling. The key challenge is to understand how regulated ROS delivery from the PETC to the Cys‐based redox machinery is organised to transmit redox signals from the environment to the nucleus. Redox changes associated with stromal carbohydrate metabolism also play a key role in chloroplast signalling pathways.

## Significance Statement

Chloroplasts are major producers of reactive oxygen species (ROS) that are important signals in the communication between the chloroplasts and the nucleus. The photosynthetic electron transport chain (PETC) has evolved to control ROs production and thus limit collateral damage while facilitating essential signalling functions. We consider current concepts of ROS production and processing in chloroplasts, highlighting new directions and gaps in our current knowledge.

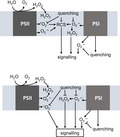

## INTRODUCTION

Chloroplasts harvest sunlight to drive the process of photosynthesis allowing the assimilation of carbon and nitrogen and the synthesis of a portfolio of primary and secondary metabolites that are the basis for plant growth and biomass production. The enzymes of carbon and ammonia assimilation and their regulatory proteins are localised in the chloroplast stroma, which bathes the thylakoid membrane system and the circular DNA of the plastome. The photosynthetic electron transport chain (PETC) not only drives photosynthetic carbon assimilation, but it also generates a repertoire of signals that link energy generation to environmental changes and nuclear gene expression. Photosynthetic metabolism is coordinated with daily and seasonal environmental variations and rhythms in physiology and development are matched to carbon availability (Román et al., 2021). Moreover, chloroplasts are important in establishing effective systemic acquired resistance, a process that confers broad‐spectrum and lasting immunity to diverse pathogens, as well as pathogen‐associated molecular patterns‐triggered immunity (PTI) and effector‐triggered immunity (ETI; Fernandez & Burch‐Smith, 2019). The ROS produced in chloroplasts play a pivotal role in this process in establishing plant immunity to pathogens (Littlejohn et al., 2021). Oxygenic photosynthesis produces large quantities of ROS in the forms of singlet oxygen (^1^O_2_), superoxide (O_2_^●−^), hydroxyl radicals (OH^**●**^) and hydrogen peroxide (H_2_O_2_) through energy or electron donation to ground state molecular oxygen. In addition, large amounts of H_2_O_2_ are generated in peroxisomes as a result of the metabolism of phosphoglycolate, which is produced by and exported from chloroplasts during photorespiration (Noctor et al., 2002). Some authors still often refer to ROS as ‘toxic by‐products of aerobic metabolism’. This blinkered view has long had the negative effect of colouring or limiting concepts regarding ROS production, accumulation, functions and fate (Mittler, 2017). The consensus of opinion has shifted to focus on the beneficial roles of ROS in many essential plant processes, particularly cell to cell communication, cell proliferation, growth and stress responses (Huang et al., 2019; Mhamdi & Van Breusegem, 2018), functions which demonstrate that ROS are essential markers or signals of living cells (Mittler, 2017; Van Breusegem et al., 2018). ROS signals have been implicated in the regulation of numerous development processes from root development (Mase & Tsukagoshi, 2021) and the transition to flowering (Huang et al., 2021) to leaf senescence (Zentgraf et al., 2022). Within this context, chloroplast‐derived ROS not only regulate supply and demand in energy metabolism (Foyer et al., 2017; Noctor & Foyer, 2016), but they also contribute to the elicitation of genetic and epigenetic responses that allow acclimation and adaptation to metabolic, developmental and environmental triggers (Mhamdi & Van Breusegem, 2018; Mittler, 2017).

^1^O_2_ and OH^**●**^ are so reactive that they interact with any biomolecules in the vicinity of their production, with potentially damaging consequences. However, they are also powerful signalling molecules that transmit signals through the products of their reactions. In contrast, O_2_^●−^ and H_2_O_2_ engage in electron transfer (redox) processes because they are capable of acting as either electron donors (**red**uctants) or acceptors (**ox**idants). Within the context of chloroplast redox biology, these include low molecular weight antioxidants such as ascorbate and α‐tocopherol, glutathione (GSH) and thiol proteins such as thioredoxin (TRX) and peroxiredoxins (PRX). In this way they can drive redox regulation and/or redox signalling (Becker, 2016; Santolini et al., 2019). We still know very little about the coordinated action of redox active molecules in chloroplasts and whether they function as a single synchronised network. Hence, chloroplasts are not only able to accommodate a changing redox landscape but have also harnessed the oxidative activity of ROS to regulate many if not all metabolic, genetic and epigenetic functions (in photosynthetic tissues), in addition to preventing uncontrolled oxidation (Noctor & Foyer, 1998; Rutherford et al., 2012).

The concept of energy conservation within the PETC is ambiguous, given that light provides a limitless supply of energy. In mitochondria, respiratory control serves to conserve glucose and prevent substrate depletion. In contrast, the term ‘photosynthetic control’ is less well defined and is used in different ways. This is partly due to the greater flexibility and dynamic nature of PETC when compared to the respiratory electron transport in mammalian mitochondria, but all definitions share a common theme of restricting PETC according to energy consumption in the chloroplast. Photosynthetic control has been classically used to delineate the specific mechanism that limits electron flow at the cytochrome *b*_6_ *f* (Cyt *b*_6_ *f*) complex, by slowing re‐oxidation of plastoquinol (PQH_2_) when lumen pH is low (Tikhonov et al., 1981). In addition, it has also been used to describe the ratio between electron flow in the presence and absence of ADP in intact chloroplasts (West & Wiskich, 1968) and the interactions with all the downstream redox processes/mechanisms that affect the operation of the PETC (Foyer et al., 2012). The pool sizes of chloroplast reductants and adenylates are small and hence they require constant turnover (Gerst et al., 1994). Thus, the network of coordinated redox regulation limits over‐reduction and over‐oxidation in the system by ensuring that energy production by the PETC is synchronised with the stromal processes that use the ATP and reducing power produced by electron transport.

The redox reactions in the PETC are coupled to downstream electron acceptors, including molecular oxygen, leading to ROS production, particularly when acceptors become limiting in the chain (Khorobrykh et al., 2020). This process, which was first demonstrated by Mehler (1951) and is thus often called the Mehler reaction, was for many decades inextricably linked to the concept of oxidative stress. ROS production by the PETC leading to photodamage is often considered to be an unavoidable consequence of the operation of electron transport carriers in an oxygen‐rich environment (Khorobrykh et al., 2020). However, as we will discuss below, the photosynthetic complexes have evolved to control and minimise electron transport to oxygen (Rutherford et al., 2012). Moreover, ROS production has been harnessed to a role in cell signalling important for plant defences against biotic and abiotic stresses (Mielecki et al., 2020).

## BRIEF HISTORICAL RECORD

**Figure 1:**
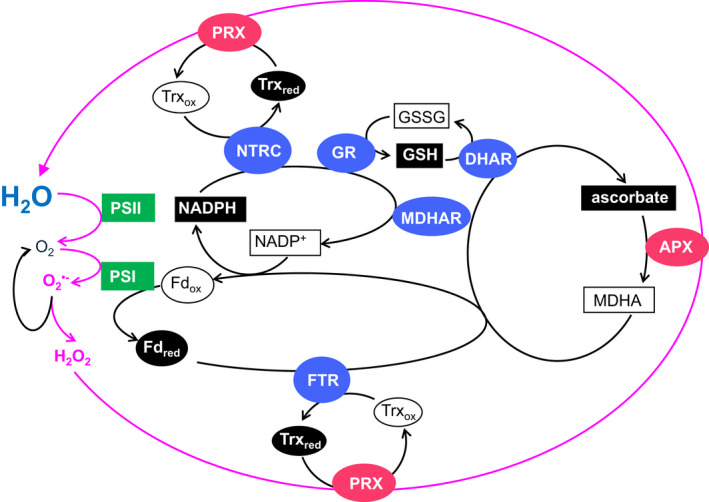
Schematic model of the water–water cycle in photosynthesis. During photosynthetic electron transport, the reduction of molecular oxygen in the Mehler reaction produces superoxide anion radicals, O_2_^●−^. O_2_^●−^ is then either directly reduced to hydrogen peroxide (H_2_O_2_) by ascorbate, or this radical can participate in a dismutation reaction to produce H_2_O_2_, a reaction that is catalysed in chloroplasts by the thylakoid and stromal superoxide dismutases. H_2_O_2_ is then reduced to water by the action of either chloroplast ascorbate peroxidases (APXs) or chloroplast 2‐Cys peroxiredoxins (PRXs). The APX reaction is the first step in the ascorbate/glutathione (GSH) cycle, which serves to regenerate ascorbate and maintain the ascorbate pool in its reduced form, using NADPH produced by the photosynthetic electron transport chain. These reactions start with the splitting of water in the PSII reaction centre and end with the reduction of O_2_^●−^ and H_2_O_2_ to water in the stroma using reducing power provided by the photosynthetic electron transport chain. The overall process, which involves successive oxidations and re‐reductions of ascorbate, peroxiredoxins (PRXs) and thioredoxins (TRX), is often referred to as the water–water cycle of photosynthesis. This cycle is crucial in preventing the accumulation of H_2_O_2_ in the stroma to levels that would inhibit the thiol‐modulated enzymes of the Calvin–Benson–Basham cycle and other pathways. White writing on black, free radicals; white writing on grey, enzymes; black writing in white boxes, redox couples in the cycle.

The past 70 years have witnessed an explosion of interest in the production and roles of ROS in chloroplasts. Following the first description of the mechanism of the reduction of oxygen in chloroplasts by Mehler (1951), it took some time for the basic mechanistic details of the formation of O_2_^●−^ by the electron transport system and the modulation of ROS accumulation by antioxidants such as ascorbate to be resolved (Allen & Hall, 1973; Asada et al., 1974). Soon after, the localisation of the ascorbate/GSH cycle in chloroplasts was described for the first time (Foyer & Halliwell, 1976), together with the proposal that GSH functions to stabilise enzymes of the Calvin–Benson–Basham cycle and that GSH may also prevent oxidation of the chloroplast ascorbic acid pool. Current understanding of this cycle is shown in Figure 1. In the same year, the first descriptions of TRX and GSH as photosynthetic regulators in chloroplasts appeared (Buchanan & Wolosiuk, 1976, 1977; Schurmann et al., 1976). The identification of the chloroplast ascorbate‐specific ascorbate peroxidases (APXs) followed (Groden & Beck, 1979; Kelly & Latzko, 1979), together with the description of the susceptibility of the enzymes of the Calvin–Benson–Basham cycle to H_2_O_2_ (Kaiser, 1979). The major site of ROS production was identified as the acceptor side of Photosystem I (PSI) (Furbank & Badger, 1983), but at this time, ROS were regarded solely as harmful by‐products of electron transport to oxygen. The first indication that ROS had important roles in plant defence and signalling came with the pioneering studies of Doke (1983), who demonstrated that O_2_^●−^ anion generation at the plasma membrane was important in plant defences against fungal pathogens. Thereafter, the roles of ROS as important signalling molecules regulating plant responses to abiotic and biotic stresses slowly emerged and current concepts regarding ROS functions in chloroplast to nucleus signalling have emerged much more recently, as discussed below.

## PHOTOSYNTHETIC ELECTRON TRANSPORT

In photosynthesis, electrons in chlorophyll are excited by light to a high energy state and a very negative redox potential (below −0.6 V; Rutherford, 1981). This excitation can be dissipated in several ways and would normally be lost as the electron returns to the ground state, releasing heat or light as fluorescence (a process that takes place within nanoseconds). However, the generation of photosynthetic power depends on transfer of this electron at an even faster rate (within picoseconds) away from a special chlorophyll pair in the reaction centre of Photosystem II (PSII) to an acceptor before it can drop back to the ground state. The final electron acceptor of PSII is plastoquinone (PQ), and this electron then passes through a series of redox centres, generally moving from more negative to more positive redox potentials (lower energy states). The energy released by this process can then be used to power metabolism. This leaves the chlorophyll in an oxidised state (lacking an electron). Earlier in evolution, photosynthetic organisms replaced this electron by exploiting chemical sources of electrons (reducing agents), such as Fe^2+^ ions or H_2_S, but cyanobacteria exploited the extraordinarily positive redox potential (capacity to accept electrons) of oxidised chlorophyll (around +1.2 V; Rappaport et al., 2002) to extract electrons from an unlimited supply, namely H_2_O, releasing O_2_ as a by‐product.

The dynamic regulation of the PETC allows continuous adjustments to accommodate changes in the availability of light and CO_2_. Photosynthetic regulation has evolved to maximise protection and to maintain a balance between energy‐producing and energy‐consuming processes. Since light is a potentially dangerous energy source and its supply is erratic, its use must be carefully managed, not least because PSII is extremely sensitive to light‐induced oxidative inactivation (Yokthongwattana & Melis, 2006). Optimising the efficiency of photosynthesis is a priority when light is limiting, allowing effective use of solar power and ensuring that metabolism is not limited by energy supply. In contrast, energy dissipation is progressively increased with increasing irradiance to prevent light‐induced inhibition (Ruban, 2016), particularly at low temperatures or under high light.

## ROS PRODUCTION

**Figure 2:**
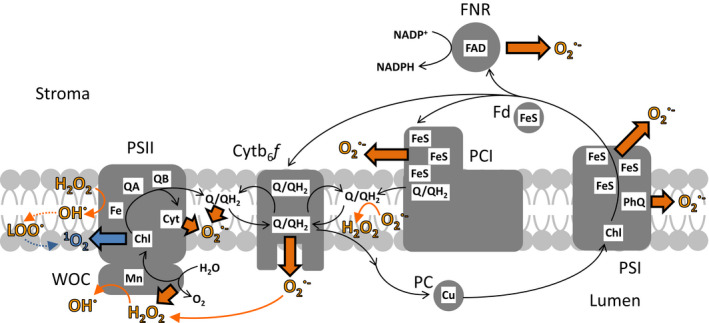
Reported and proposed sites of free radical generation in the photosynthetic light‐harvesting and electron transport apparatus. Light excitation of chlorophyll at PSII and PSI drives electron transport (shown in black and white arrows), but in the absence of acceptors, excitation may be transferred to O_2_ at the PSII reaction centre forming the singlet oxygen radical (^1^O_2_). This process has also been measured in purified PSI (probably from the antennae) and may occur on photodamage to the photosystem. During PET, donation of a single electron to the two electron carrier plastoquinone (PQ) at the cytochrome *b*_6_ *f* (Cyt *b*_6_ *f*) complex results in formation of the semiquinone radical, which reduces oxygen to superoxide (O_2_^●−^). This process also happens during electron transfer from the single electron carrier ferredoxin (Fd) to NADP^+^ by Fd:NADP(H) reductase (FNR) and could potentially occur at photosynthetic complex I (PCI). Reduction of O_2_ can also occur by electron donation (i) from the cytochrome *b*_559_ cofactor (Cyt) of PSII, (ii) from reduced plastoquinone (PQH_2_) and (iii) at PSI from phyloquinone (PhQ) to O_2_ in the membrane phase and from the FeS clusters to O_2_ in the stroma. In solution, O_2_^●−^ is readily dismutated to H_2_O_2_, while in the membrane phase electron donation from PQH_2_ catalyses this reaction. H_2_O_2_ is also produced directly during incomplete oxidation of water to O_2_ at the water oxidation complex. In the presence of metal centres, such as Fe or (much less efficiently) Mn, H_2_O_2_ is converted to hydroxyl radicals (OH^•^), which readily generate carbonyl radicals (D'Alessandro et al., 2020), of which only lipid peroxide radicals (LOO^•^) are shown here. OH^•^ is also thought to arise from reduction of peroxides formed when O_2_^●−^ interacts with free metal centres, principally the non‐heme Fe in PSII. Dotted lines indicate several intermediate steps. Free radical generation involving excitation transfer is shown in blue, radical generation involving electron transfer is shown in orange. Several light‐harvesting and cyclic electron flow components are omitted for clarity. Note the different compartments (stroma, lipid phase and thylakoid lumen) in which radicals are produced by different cofactors.

An overview of ROS production by the PETC is shown in Figure 2. There are two basic mechanisms by which ROS are formed. The first is transfer of an excited electron spin state from chlorophyll to O_2_ (normally in the triplet state) to form the highly reactive ^1^O_2_. This occurs when excited chlorophyll (originally in the singlet state) drops down to the lower energy triplet state or following a charge recombination reaction at PSII due to the lack of an electron acceptor (Krieger‐Liszkay, 2005). Triplet chlorophyll has an even longer half‐life (microseconds) than excited singlet state chlorophyll (nanoseconds), which gives ample time for excitation transfer to triplet oxygen. The great majority of ^1^O_2_ generated in the photosynthetic membrane has its origin at the PSII reaction centre. The PSI reaction centre is buried deep in the protein, and it is thought that this screens it from O_2_, preventing excitation transfer and the formation of ^1^O_2_ (Setif et al., 1981). It has been reported that the low rates of ^1^O_2_ formation actually detected at PSI originate from excitation transfer in the light‐harvesting complexes surrounding the PSI core (Cazzaniga et al., 2016).

Secondly, electrons can also be transferred directly to O_2_, forming O_2_^●−^. The midpoint redox potential for O_2_/O_2_^●−^ in aqueous solution is around −160 mV (Wardman, 1989), meaning that it is energetically favourable for O_2_ to gain an electron from many reduced components of the PETC. The predominant factors determining whether this occurs are the lifetime of the reduced cofactor and its accessibility to O_2_. It should therefore be of no surprise that PSI, the point at which electrons are transferred out of the membrane environment to soluble acceptors, is the predominant site of O_2_^●−^ generation in chloroplasts. This reaction may be exacerbated when there is a lack of available electron acceptors, such as when CO_2_ concentrations limit the consumption of electrons by the Calvin–Benson–Basham cycle due to stomatal closure. However, it is likely that photorespiration becomes an effective electron sink in these circumstances (Fernie & Bauwe, 2020; Wada et al., 2020). Indeed, photorespiration, which produces huge amounts of H_2_O_2_, has been considered to be a stepping stone towards the evolution of oxygenic photosynthesis in the first instance and later C_4_ photosynthesis (Fernie & Bauwe, 2020).

**Figure 3:**
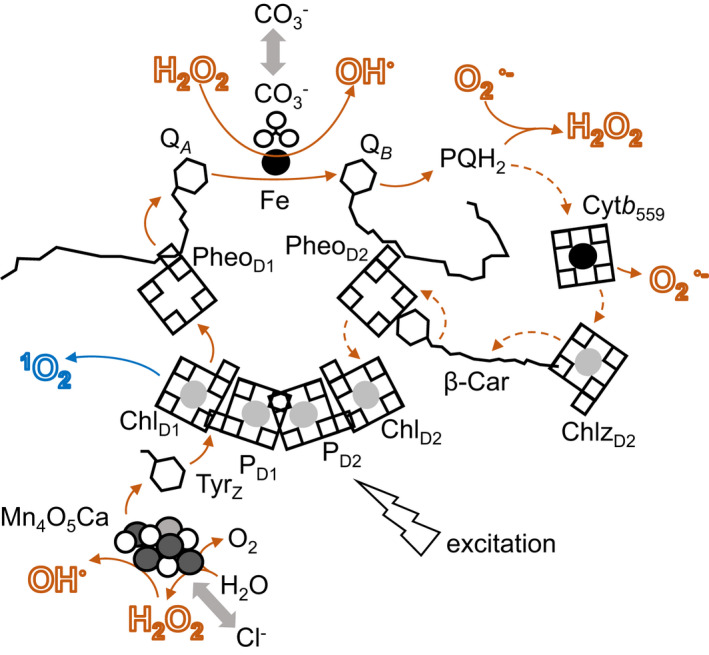
ROS production in the PSII core. Redox active cofactors of PSII are represented in approximate orientation. Boxes represent pyrroles, forming chlorophyll (Mg shown as light grey circle inside), pheophytin (empty) and heme (Fe shown as black circle inside) tetrapyrrole rings. Carotene (car) and quinone (Q) molecules and the side chain of a specific tyrosine residue are shown as C skeletons. Electron transfer is shown as orange arrows, excitation transfer is shown in blue. Excitation of the P680 reaction centre in PSII results in electron donation from the special pair of chlorophylls (P_D1_ and P_D2_) to pheophytin in the D1 subunit of PSII (Pheo_D1_). From here electrons pass to the quinone acceptor Q_*A*_ and then to Q_*B*_. In the absence of quinone acceptors, backflow of electrons to P680 can result in transfer of excitation from triplet chlorophyll to form singlet oxygen. In this case, the reduced state of Q_*A*_ results in bicarbonate release from the non‐heme Fe, and this shifts the QA redox potential to limit this backflow of electrons. The non‐heme iron is also presumed to be the source of OH^●^ production in the lipid phase by PSII. PQH_2_ released from PSII can reduce O_2_^●−^ to H_2_O_2_. The proposed cyclic mode of electron transport in PSII, with electrons passing from Cyt *b*_559_ to the oxidised P680, is shown in dashed lines. Reduced Cyt *b*_559_ is considered a potential electron donor to O_2_^●^, forming O_2_^●−^ in the membrane lipid phase. Electrons lost from P680 are extracted from the Tyr_*Z*_ side chain, which in turn acquires them from the Mn_4_O_5_Ca cluster of the water‐oxidising complex (WOC). On full oxidation, the WOC splits water. Loss of Cl^−^ ions from close to the Mn_4_O_5_Ca cluster is related to H_2_O_2_ production following extraction of just two electrons from H_2_O. Free Mn and Fe ions released from damaged photosystems can act as Fenton catalysts.

O_2_^●−^ production is possible at multiple points in the electron transport chain, depending on metabolic status. The literature suggests that in addition to the Mehler reaction at PSI, O_2_^●−^ and H_2_O_2_ can form within PSII when the electron transport system is reduced (Tiwari & Pospisil, 2009). A more detailed picture of proposed ROS production at PSII is shown in Figure 3. In addition, the PQ pool (Khorobrykh et al., 2015), the plastid terminal oxidase (PTOX) (Heyno et al., 2009) and the Cyt *b*_6_ *f* complex (Baniulis et al., 2013) have the potential to reduce oxygen to O_2_^●−^. The reduction of molecular oxygen at PSI in the Mehler reaction initiates the pathway of pseudocyclic electron flow. This is classically considered to be an unavoidable consequence of electron transport in an aerobic environment. In this process, O_2_^●−^ radicals are generated at the stromal surface of the thylakoid membrane and rapidly converted to H_2_O_2_ by the action of the thylakoid O_2_^●−^ dismutases (SODs). H_2_O_2_ can then be reduced to water by chloroplast APXs and 2‐Cys PRXs (König et al., 2002). The overall process became known as the water–water cycle because two electrons are used to produce H_2_O_2_ and two more are required to metabolise H_2_O_2_ to water, allowing the dissipation of excess excitation energy and electrons (Figure 1; Asada, 2000). The ascorbate‐dependent water–water cycle can account for up to 30% of the electron flux (Miyake, 2010). As such the water–water cycle provides an alternative electron sink that protects PSII from inhibition and still supports ATP production (Neubauer & Yamamoto, 1992). However, because this process is saturated at relatively low irradiances, authors have more recently suggested that the water–water cycle can make only a minor contribution to thylakoid acidification and the control of PSII activity (Driever & Baker, 2011). The thylakoid and stromal antioxidant systems modulate the accumulation of ROS produced in photosynthesis and thus regulate environmental sensing (Fichman et al., 2019) through the modulation of oxidative signals that are exported from the chloroplasts to the nucleus (Exposito‐Rodriguez et al., 2017; Gollan & Aro, 2020).

During photosynthesis, ROS are predominantly produced at the PSII reaction centre (^1^O_2_) and the PSI acceptor side (O_2_^●−^), but production of free radicals has been measured from several other sites in the light‐harvesting and electron transport apparatus. In some cases, these sites could contribute a significant proportion of ROS generated (Kozuleva & Ivanov, 2016), in others ROS generation has been proven for isolated components *in vitro* (Baniulis et al., 2013) but remains to be detected in vivo. It is also possible to speculate on other potential sites of ROS generation in the PETC, based on cofactor type, redox potential and exposure to the solvent. The high reactivity of ROS, especially ^1^O_2_, means that their downstream impact through signalling cascades may be transduced by the reaction products of secondary interactions, in turn dependent on the molecules in the immediate environment when they are first generated. ROS generation by the thylakoid membrane can be categorised in terms of the location in the chloroplast into which ROS are released, as illustrated in Figure 2.

## STROMAL SIDE ROS PRODUCTION

All the cofactors on the reducing side of PSI are able to reduce O_2_ in the stroma, which can potentially be destructive for the FeS cluster cofactors in the electron transport chain (Sonoike et al., 1995). In PSI, excited electrons pass from the reaction centre special pair through other chlorophyll molecules and a phylloquinone (PhQ) to a chain of three 4Fe–4S clusters at the stromal side of the complex (Ben‐Shem et al., 2003; Shimakawa & Miyake, 2018). The terminal FeS clusters (FA/FB) are oxidised by O_2_ when the soluble electron carrier protein ferredoxin (Fd) is not available in an oxidised state. Moreover, the PhQ molecules at the A1 sites may also reduce oxygen (Kozuleva et al., 2014). Although the FB cluster is the direct electron donor to Fd (Diaz‐Quintana et al., 1998), electrons reside largely on the FA site that is further from the stromal surface (Shinkarev et al., 2000). This is because FB has a more negative redox potential than FA (Heathcote et al., 1978), making electron transfer in the final step of the cascade through PSI ‘uphill’, and therefore limiting flux to O_2_ in the absence of Fd. Hence, the reactivity of FeS components such as FB and Fd with oxygen is decreased by redox tuning and protein shielding. Although Fd, which contains a 2Fe–2S cluster, is a relatively poor electron donor to O_2_ in comparison to FA/FB (Kozuleva & Ivanov, 2016), it is still sometimes described as a source of electrons for O_2_^●−^ generation; for example see Allen (1975). This finding is usually based on increased O_2_^●−^ generation following addition of Fd to active PSI preparations or thylakoids. In this case the unphysiologically high Fd:PSI ratios might give the false impression that Fd is a more effective O_2_ reductant than the PhQ and FA/FB clusters of PSI. The two electron reduction of NADP^+^ to NADPH is catalysed by Fd:NADP(H) reductase (FNR) using a flavin (FAD) cofactor. Discrepancies between supply of reduced Fd (a one electron carrier) and NADP^+^ (a two electron acceptor) can leave this cofactor in a single electron reduced (semiquinone) state, which also readily reduces O_2_ (Kozuleva et al., 2016).

In addition to the proven O_2_^●−^ generation at PSI, there are other potential sources of O_2_^●−^ on the stromal side of the membrane. The structure of the cyanobacterial NDH complex (more recently termed photosynthetic complex I [PCI]) has recently been solved (Laughlin et al., 2019; Schuller et al., 2019). This complex is also present in the chloroplasts of angiosperms (Corneille et al., 1998; Sazanov et al., 1998) and is a homologue of respiratory complex I, a notorious generator of O_2_^●−^ on the n‐side of the membrane (Hernansanz‐Agustín et al., 2017) (stromal side in chloroplasts). The terminal FeS cofactor in PCI, N0, is very solvent‐exposed (Schuller et al., 2019), and in the purified complex it has an extremely negative redox potential (below approximately −550 mV; Richardson et al., 2021). If this is the *in vivo* configuration of the FeS cluster, it is hard to see how electron donation to O_2_ could be avoided under conditions of abundant reduced stromal electron donors and a reduced PQ pool. This raises the possibility that PSC1 could make a large and as yet uncharacterised contribution to ROS generation in chloroplasts under specific redox conditions. Although this complex is only present at low abundance in most species (Shikanai & Yamamoto, 2017), specific cell types with high ATP demand (e.g. in C4 plants) can contain much higher levels (Darie et al., 2006; Ishikawa et al., 2016; Munekage et al., 2010), so there may be situations where NDH/PCI makes a significant contribution to O_2_^●−^ generation.

## LIPID PHASE ROS PRODUCTION

^1^O_2_ is predominantly generated by direct energy transfer from the triplet excited state of chlorophyll to molecular oxygen (Figure 3) and is so reactive that diffusion from the PSII reaction centre is unlikely given its very short half‐life (nanoseconds). The occurrence of photosensitisers such as triplet excited chlorophylls occurs mainly in the PSII reaction centre (D'Alessandro & Havaux, 2019; Pospisil, 2016). It is thought that ^1^O_2_ is inevitably produced by photosynthesis even in low light conditions, albeit at greatly decreased rates in comparison to high light conditions (Fufezan et al., 2002). In contrast to H_2_O_2_, ^1^O_2_ is a powerful oxidant that reacts rapidly with macromolecules in its vicinity, resulting in oxidation, which is often referred to as ‘damage’, or perhaps more accurately ‘collateral damage’ (Santolini et al., 2019). The peroxidation of lipids in the polyunsaturated fatty acid‐rich thylakoid membranes produces lipid hydroperoxides, aldehydes and reactive electrophile species, which are further broken down to prevent accumulation that would inevitably lead to cell toxicity (Mano, 2012). To limit these effects of ^1^O_2_ in the lipid phase, thylakoid membranes are rich in vitamin A, carotenoids and xanthophylls, which are incorporated into chlorophyll‐containing complexes in high concentrations. Prenyl lipids such as α‐tocopherol, PQ‐9 and carotenoids not only quench ^1^O_2_ but they also efficiently scavenge O_2_^●−^. It is possible that α‐tocopherol is sufficiently close to Pheo_D1_ and non‐heme iron to protect the amino acids of the D1 protein against oxidative damage (Kumar et al., 2021).

An array of antioxidant molecules is available in the lipid phase of the membrane, where de‐epoxidation of violoxanthin to zeaxanthin is critical to prevent lipid peroxidation (Havaux & Niyogi, 1999). The vulnerability of the D1 and D2 proteins to oxidative modifications means that each reaction centre has to be repeatedly rebuilt, even under optimal light conditions, with rates estimated between every 7 h and every 20 min, depending on the light intensity. The oxygen‐evolving complex is also prone to ^1^O_2_‐mediated damage/modification (Henmi et al., 2004). Low rates of ^1^O_2_ production have been detected at PSI and are thought to originate from the antenna (Cazzaniga et al., 2016). Although these likely make a minimal contribution to lipid phase ROS production under optimal conditions, this may be exacerbated upon damage of PSI, when destruction of the FeS clusters is followed by damage to antenna proteins.

The presence of significant quantities of O_2_^●−^ within the thylakoid membrane has been described (Kozuleva & Ivanov, 2016). This is possible because the concentration of O_2_ in the lipid bilayer is assumed to be high during oxygenic photosynthesis, although it has a midpoint potential here of −550 to −600 mV (Afanas'ev, 1989), limiting the pool of potential reductants. Within the thylakoid membrane lipid phase, O_2_ reduction by reduced PQ, i.e. PQH_2_, may be important. PQH_2_ and the plastosemiquinone are relatively stable in the presence of oxygen (Hasegawa et al., 2017). However, PQH_2_ was found to reduce oxygen directly to H_2_O_2_, by further reducing O_2_^●−^ (Borisova‐Mubarakshina et al., 2018). PSII is also a potential stromal source of O_2_^●−^ radicals when the PQ acceptor pool is fully reduced, for example when electrons suddenly flood the system during sunflecks. Of the PSII cofactors that function in electron transfer from the reaction centre to PQ, only pheophytin (Pheo−) has a redox potential capable of reducing O_2_ in the lipid environment, but it has such a short lifetime it is unlikely to contribute (Rappaport et al., 2002). Another potential mechanism by which O_2_ could be reduced in the lipid phase at PSII is via the low potential form of Cyt *b*559 at PSII (Khorobrykh, 2019), but this is very unlikely unless PSII functions are severely perturbed. PSI is also a source of lipid phase O_2_^●−^. In fact, a report by Kozuleva et al. (2021) suggests that at high irradiance, O_2_^●−^ production from the PhQ in PSI exceeds even that from the FeS clusters on the stromal side. The production of dangerous OH^•^ radicals within the lipid phase is also possible. This can occur via reduction of a peroxide generated when O_2_^●−^ interacts with a metal centre at PSII, probably the non‐heme iron (Pospisil et al., 2004). These highly reactive ROS rapidly react with lipids to form peroxidation products such as lipid hydroperoxides, which can decompose to a range of radical species, and some long‐lived intermediates such as lipid hydroperoxides (Pospisil, 2016), which are potential signalling molecules. Moreover, some of the high energy intermediates are capable of forming triplet carbonyls, which might be capable of energy transfer to O_2_, forming ^1^O_2_. The contribution of this pathway to ^1^O_2_ production *in vivo* remains to be quantified. Protection against radical species such as ^1^O_2_ and hydroxyl radicals is mediated by vitamin A and vitamin E molecules. Carotenoids can also scavenge these radicals by adduct formation with the radical and loss of an electron to give either a carotenoid radical cation (Liebler & McClure, 1996) or a carotenoid radical (Woodall et al., 1997).

## LUMEN SIDE ROS REDUCTION

Oxygen reduction at the luminal side occurs at Q_p_ (the quinone binding site on the positive side of the membrane) of the Cyt *b*_6_ *f* complex (Taylor et al., 2018) by electron donation to O_2_ from either FeS or a *b*L heme cofactor. The frequency of these reactions is thought to be decreased by the dimer organisation of the *b*_6_ *f* complex, which provides alternative electron transport pathways between monomers (Rutherford et al., 2012). O_2_^●−^ formation at the Cyt *b*_6_ *f* complex appears to be much more active than in the homologous respiratory complex III, which is also a dimer (Baniulis et al., 2013). Although this is a relatively small amount of ROS compared to that generated at PSI, it is presumed to be on the opposite side of the membrane and therefore might induce alternative signalling responses, as discussed later.

On the luminal side of the membrane, direct generation of H_2_O_2_ is also possible at the water oxidation complex of PSII, when only two electrons are extracted from water. This appears to be related to loss of chloride from close to the Mn_4_O_5_Ca cluster, which normally helps regulate H_2_O access to the metal centre (Fine & Frasch, 1992). Single electron reduction of H_2_O_2_ forms the more dangerous OH^•^ radical in the Fenton reaction, which can be catalysed by free Fe or (much more slowly) Mn cofactors within the electron transport chain (Pospisil et al., 2004; Šnyrychová et al., 2006), such as those in the water oxidation complex.

## THIOREDOXIN‐DEPENDENT CONTROL OF CHLOROPLAST METABOLISM

**Figure 4:**
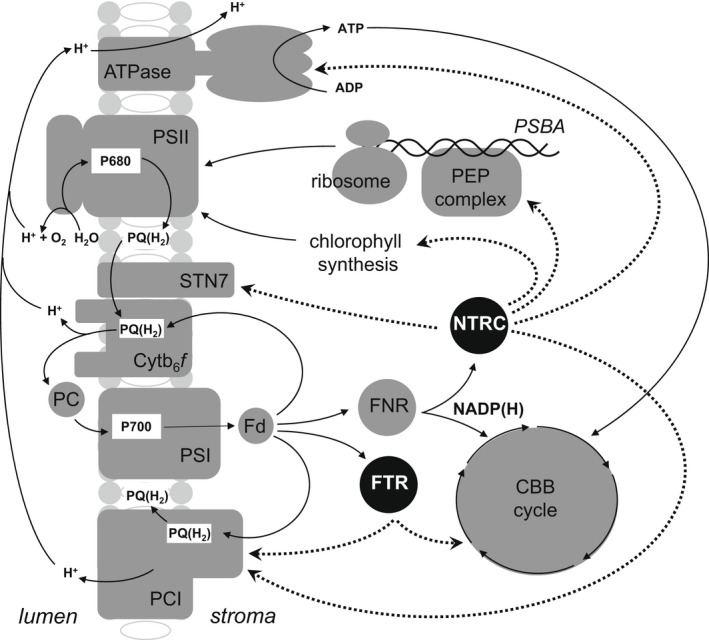
Schematic model of the regulation of photosynthetic electron transport by the chloroplast thioredoxin system. The photosynthetic electron transport chain drives electrons from PSII through the cytochrome *b*_6_ *f* (Cyt *b*_6_ *f*) complex to PSI. PSI uses these electrons to reduce ferredoxin (Fd). This Fd can then be used (i) to reduce NADP to NADPH, a reaction that is catalysed by ferredoxin:NADP reductase (FNR), (ii) to reduce oxidised thioredoxin (TRX) to its reduced form in a reaction catalysed by ferredoxin:NADP reductase (FTR) or (iii) to transfer electrons back to either the Cyt *b*_6_ *f* complex or NDH/PCI. TRX can also be reduced by an NADPH‐dependent thioredoxin reductase (NTRC) pathway, which is also capable of directly reducing thiol disulphides on some proteins. The FTR and NTRC pathways have been reported to oppositely regulate the NDH/PC‐1 complex depending on conditions. Both the FTR and NTRC pathways can also activate certain carbon assimilation enzymes but under rather different light conditions. The NTRC pathway is responsible for the regulation of (i) ATPase, (ii) chlorophyll synthesis, (iii) the expression of chloroplast genes (such as *PsbA*, which encodes the D1 protein) through the plastid‐encoded polymerase (PEP) complex and chloroplast translation and (iv) D1 excision and repair pathways. NTRC is also required for the regeneration of the 2‐Cys peroxiredoxins and the transfer of electrons to the thylakoid lumen via the membrane‐anchored CcdA/HCF164 system, in order to regulate enzymes such as the state transition 7 (STN7) kinase.

Reductive signals are mainly generated by the flow of electrons from the PETC into the TRX network, which regulates nearly all chloroplast processes from biogenesis and assembly of chloroplast machinery to the functional operation of photosynthesis through the modulation of light harvesting, carbon fixation in the Calvin–Benson‐Basham cycle, protection of thylakoid membranes, plastome transcription and translation and chlorophyll synthesis (Nikkanen & Rintamäki, 2019). Chloroplasts house two fd/TRX systems (Figure 4): one in which TRX is reduced by ferredoxin‐thioredoxin reductase (FTR) and one in which TRX is reduced by NADPH‐TRX reductase (NTRC). Although these systems serve rather different functions, they work together to ensure the redox regulation of the multiple chloroplast TRX (f, m, x, y, z) and TRX‐like proteins (Balsera et al., 2014; Geigenberger et al., 2017; Nikkanen & Rintamäki, 2019). Both systems are able to reduce redox‐regulated chloroplast proteins but with different efficiencies, such that the specific functions of each system cannot be compensated by the other system (Nikkanen & Rintamäki, 2019).

The light‐induced reductive activation of chloroplast proteins is reversed by the 2‐Cys PRX system, which serves as a rapid electron sink for the thiol network (Ojeda et al., 2018; Vaseghi et al., 2018). The 2‐Cys PRXs are remarkably efficient thiol peroxidases that are required for the dark‐induced oxidation of Calvin–Benson–Basham enzymes, although they are less effective with regard to oxidation of other thiol‐regulated chloroplast proteins such as the CF1‐γ subunit of the chloroplast ATPase (Carrllio et al., 2016; Ojeda et al., 2018). Specific atypical TRXs, such as ACHT1‐4 and TRXL2, transfer oxidative equivalents from photosynthetically produced H_2_O_2_ to target proteins (Yokochi et al., 2021; Yoshida et al., 2018). Such results indicate that the chloroplast thiol‐modulated enzymes are not rapidly oxidised by H_2_O_2_ alone because the dark‐dependent oxidation of chloroplast enzymes is delayed in mutants lacking 2‐Cys PRXs (Ojeda et al., 2018). The rate of 2‐Cys PRX oxidation is however controlled by the flux of electrons through the water–water cycle (Figure 1).

## THIOREDOXIN‐DEPENDENT CONTROL OF ELECTRON TRANSPORT PROCESSES

Numerous chloroplast elongation factors, chaperones and kinases are subject to redox modulation to provide essential control over gene expression, protein conformation and post‐translational modification (PTM) in the processes of PSII repair. For example, oxidation can limit the replacement of damaged D1 proteins because the EF‐Tu translation factor, which is required for delivery of aminoacyl‐tRNAs to the ribosomes, is inactivated by oxidation of the susceptible Cys82 residue (Jimbo et al., 2018). This regulation ensures that there is an appropriate balance between the supply and demand for essential photosynthetic components and prevents the potentially dangerous accumulation of intermediates, such as the photodynamic metabolites in tetrapyrrole and chlorophyll biosynthesis pathways. There are many examples of redox regulation of chloroplast translation such as the formation of the Nac2–RBP40 complex, which is a chloroplast mRNA translation factor that is required for the synthesis of PSII D2 protein in green algae such as *Chlamydomonas reinhardtii* (Sun & Zerges, 2015). This high molecular weight complex contains the RNA stabilisation factor Nac2 and the translational activator RBP40, which are linked by an intermolecular disulphide bridge that is regulated by the NTRC system (Sun & Zerges, 2015).

Plastid TRX isoforms belonging to the x‐, y‐ and z‐types serve a range of functions such as reduction of PRX, thiol peroxidases and methionine sulphoxide reductases. In particular, the presence of TRXz as part of the plastid‐encoded RNA polymerase (PEP) suggests the possibility of redox control of chloroplast gene expression. However, the phenotype of the *trxz* mutant was restored by redox‐insensitive variants of TRXz, suggesting that redox regulation plays a minor role in the regulation of PEP‐dependent transcription (Wimmelbacher & Börnke, 2014). Chloroplast gene transcription is facilitated by two distinct types of RNA polymerases in the chloroplasts of higher plants: PEP and a nucleus‐encoded RNA polymerase (NEP). NEP is encoded by two nuclear genes, rpoTp and rpoTmp, in *Arabidopsis thaliana*. In contrast, PEP is a bacteria‐type RNA polymerase composed of four core subunits and a promoter‐recognising subunit (σ factor), as well as 12 nuclear‐encoded PEP‐associated proteins (PAPs) (Pfannschmidt et al., 2015). Mutations in most of the *pap* genes result in shared albino/ivory phenotypes because of epistasis (Pfannschmidt et al., 2015). All PAP components are required for the stability of the complex and hence photosynthesis is only possible when all the functional components are present (Liebers et al., 2018).

The genes encoding σ factors provide the necessary promoter specificity to PEP, allowing the nucleus to regulate chloroplast gene transcription in response to environmental and developmental cues. PEP and a set of PAPs are involved in the regulation of DNA and RNA metabolism in chloroplasts. TRXz and the plastid fructokinase‐like proteins FLN1 and FLN2 are intrinsic subunits of the PEP enzyme of chloroplasts (Schröter et al., 2010). These proteins and other PAPS such as PAP6/FLN1, PAP10/TRXz and PAP12/pTAC7 have potential, but as yet unproven, roles in redox regulation. Other components such as PAP4/FSD3 and PAP9/FSD2 are iron SODs (Zhang et al., 2020). SODs, which are not often found bound to protein complexes, catalyse the dismutation of O_2_^●−^ radicals into H_2_O_2_, and it is likely that this is their function in the PEP complex. If this is the case, the stromal APXs (sAPXs) may be sufficient to remove the H_2_O_2_ produced by these enzymes. Alternatively, TRXz may protect the transcriptional machinery from H_2_O_2_‐mediated oxidation (Pfannschmidt et al., 2015). TRXz is reduced by NTRC (Yoshida & Hisaboria, 2016) and the redox regulation of PEP transcription by TRXz remains a possibility (Arsova et al., 2010). Moreover, the redox regulation of plastid redox insensitive 2 (PRIN2), which is essential for full PEP activity, has been demonstrated (Hernández‐Verdeja et al., 2020). The light‐mediated reduction of PRIN2 results in conversion of the dimeric, inactive form of the protein to the active monomeric form (Díaz et al., 2018). Although TRXz and TRXf1 are able to reduce the PRIN2 protein *in vitro*, there is no evidence for the functional operation of these TRXs in regulating the activity of the PEP machinery *in vivo*. Other systems may operate to regulate chloroplast translation in response to oxidative signals. For example, the expression of late embryogenesis abundant 5 (LEA5) protein is induced by oxidative stress but not by light (Karpinska et al., 2022). LEA5 plays an important in the downregulation of photosynthesis in Arabidopsis plants exposed to stress by regulating organellar translation. Expression of this protein leads to inhibition of translation in chloroplasts but conversely stimulates translation in mitochondria (Karpinska et al., 2022).

The NTRC and FTR systems serve different regulatory functions in photosynthesis (Figure 4). The NTRC system regulates photosynthetic reactions during dark/light transitions and under low light conditions. In contrast, the FTR system is responsible for redox regulation under high light intensities that do not limit photosynthesis (Nikkanen et al., 2018, Guinea Diaz et al., 2019). Arabidopsis NTRC mutants have poor light use efficiency (Carrllio et al., 2016). Conversely, the overexpression of NTRC increases photosynthetic activity (Guinea Diaz et al., 2019; Nikkanen et al., 2016) and improves light use efficiency particularly under shade conditions (Guinea Diaz et al., 2019; Nikkanen et al., 2018; Nikkanen & Rintamäki, 2019).

The regulation of cyclic electron flow through TRXm is mediated by NTRC (Courteille et al., 2013). This pathway undertakes the redox regulation of the NDH/PCI complex and the ATPase, as well as the regulation of state transitions (Nikkanen et al., 2018), as illustrated in Figure 4. Moreover, M‐Type TRXs bind to the proton gradient regulation 5 (PGR5)‐like photosynthetic phenotype 1 (PGRL1) protein, forming a disulphide‐linked complex that regulates the PGR5/PGRL1‐dependent pathway of cyclic electron flow (Okegawa & Motohashi, 2020; Wolf et al., 2020). In this way decreased PGR5/PGRL1‐dependent PQ reduction may be downregulated by complex formation. Conversely, oxygen reduction at PSI is activated by the TRX system, suggesting the operation of TRX‐dependent inverse controls of the cyclic and pseudocyclic pathways of electron flow.

Thiol‐dependent regulation of the ferredoxin binding site on the NDH/PCI complex is thought to allow dynamic control of the ferredoxin:PQ oxidoreductase activity of the complex in response to changing light conditions. As discussed above, TRXm4 regulates the PGR5/PGRL1‐dependent pathway of cyclic electron flow and is likely to decrease interactions between the NDH and PCI complexes (Courteille et al., 2013). In general, NTRC‐dependent reactions serve to enhance photosynthetic activity because they are most active at lower light intensities, where for example NDH/PCI amplification of the proton motive force would be most advantageous. By contrast, regulation through the Fd/TRX system is expected to work at higher light intensities, where NDH/PCI activity will be less beneficial (Nikkanen & Rintamäki, 2019). By regulating activity of NDH/PC1, thiol reduction reactions control the rate by which the proton motive force accumulates, but they also help control its dissipation through regulating activity of the γ‐subunit of the chloroplast ATPase (Schwarz et al., 1997) for which TRX binding to the protein is also important (Stumpp et al., 1999). NTRC is reported to be the dominant regulatory mechanism for the ATPase, although TRXf can partially rescue the loss of NTRC in a dose‐dependent manner (Guinea Diaz et al., 2019).

Several thiol‐based regulatory mechanisms of PETC remain poorly understood or are controversial or speculative. The PGR5 protein has been identified as a direct interaction partner of NTRC (Nikkanen et al., 2018), although how this relates to the TRXm regulation of PgrL1 (Okegawa & Motohashi, 2020; Wolf et al., 2020) needs to be clarified. Cyanobacteria, algae, bryophytes, pteridophytes and gymnosperms possess additional, regulatory acceptor proteins at PSI, the flavodiiron (Flv) proteins (Allahverdiyevaa et al., 2013), which catalyse the Fd‐dependent reduction of O_2_ to water (Sétif et al., 2020). This may be of significant advantage in preventing acceptor limitation and therefore ROS production at PSI. However, unregulated Flv activity could result in a competitive sink to the Calvin–Benson–Basham cycle. Thiol reduction may represent a logical mechanism for the regulation of Flv proteins (Alboresi et al., 2019). The timeframe of Flv activation (1 sec, followed by deactivation after approximately 10 sec following transfer to light) supports this view (Gerotto et al., 2016). Flv sequences have several conserved potential Cys‐Cys target pairs for either NTRC or TRX, but the only structure available to date is incomplete and contains no disulphide bridges (Borges et al., 2019), hence a definitive assignment is lacking.

The reducing power of the TRX system is transferred to the thylakoid lumen via the membrane‐anchored CcdA/HCF164 system. This transthylakoid thiol‐reducing pathway inactivates thylakoid proteins such as the serine/threonine kinase called state transition 7 (STN7) under high light conditions (Ancin et al., 2019). STN7 phosphorylates the mobile population of the light‐harvesting chlorophyll *a*/*b* binding complex II (LHCII) that captures light and transfers energy to the reaction centres. The activation state of STN7 is regulated by the Cyt *b*_6_ *f* complex, in response to changes in the balance between the oxidised (PQ) and reduced (PQH_2_) forms of PQ (Rochaix, 2014). STN7 is activated when PQH_2_ binds to the Q_p_ site of the Cyt *b*_6_ *f* complex in situations where PSII receives more excitation energy than PSI (State 1; Bellafiore et al., 2005). Following phosphorylation, LHCII migrates from PSII to PSI, increasing the PSI antenna size and rebalancing the excitation energy between PSII and PSI (State 2; Shapiguzov et al., 2016). Phosphorylation of LHC components is also considered to serve signalling functions. For example, infected cells may monitor the progression of infection through the phosphorylation of LHCB5 in a light‐dependent manner because the high light intensity‐induced phosphorylation of LHCB5 was shown to activate a ROS burst that enhances resistance to the blast fungus *Magnaporthe oryzae* in rice (*Oryza sativa*) (Liu et al., 2019).

STN7 has a transmembrane helix linking the catalytic kinase domain on the stromal side with its N‐terminus containing two conserved Cys residues in the luminal domain (Bergner et al., 2015). A lumen‐localised TRX called lumen thiol oxidoreductase1 (LTO1) interacts with luminal domains of STN7 (Wu et al., 2021). Interactions between the conserved Cys residues in the luminal domains of STN7 and LTO1 maintain STN7 in the oxidised state that is required for kinase activity during state transitions. LTO1 is part of the thiol‐oxidising pathway that catalyses disulphide bond formation (Du et al., 2015). It contains a TRX‐like domain with a similar activity to DsbA and vitamin K epoxide reductase (VKOR), which transfers electrons from TRX to the final acceptor (vitamin K epoxide) in PSI (Onda, 2013). In summary, when PSI is reduced relative to PSII (State 1), STN7 is inactivated by extraction of electrons to form a disulphide bond in the luminal domain and sequential transfer of these electrons to the TRX‐like domain and then to the VKOR domain of LTO1 (Wu et al., 2021).

**Figure 5:**
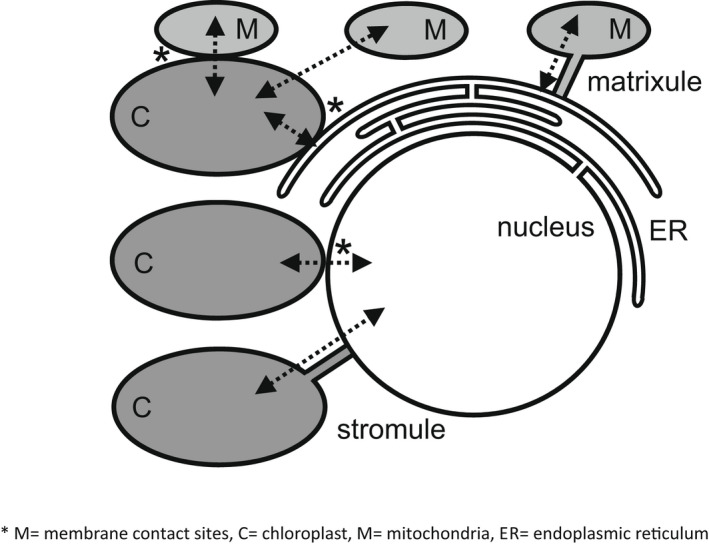
Interactions between chloroplasts and other cellular compartments through organelle protrusions and membrane contact sites. Organelle–organelle interactions in cells can occur by the formation of membrane contact sites between organelles for example with the endoplasmic reticulum (ER), or by the formation of tubular structures by one organelle. Mitochondria (M), chloroplasts (C) and peroxisomes can form tubular structures. Stromules produced by chloroplasts have a number of functions, particularly in establishing contact with the nucleus. Mitochondria form matrixules. Peroxisomes form peroxules in the vicinity of chloroplasts, mitochondria and the nucleus. These structures are considered to mediate communication between organelles by enabling exchange of signalling molecules.

NTRC is also important in chloroplast biogenesis by exerting redox control over the activities of enzymes catalysing chlorophyll biosynthesis (Richter et al., 2018). For example, the NTRC‐dependent 2‐Cys PRX activity was shown to be important in the regulation of Mg‐protoporphyrin monomethyl ester cyclase activity (Stenbaek et al., 2008). Moreover, NTRC regulates the formation of stromules (Figure 5) in response to light‐dependent changes in the redox state of the chloroplast (Brunkard et al., 2015). The generation of stromules, which are stroma‐filled tubular extensions of the chloroplast envelope containing stromal proteins, provides a potential mechanism that facilitates signal transduction between organelles, such as the nucleus, plasma membrane, endoplasmic reticulum and other plastids (Hanson & Hines, 2018).

## ROS AS RETROGRADE SIGNALS

The tolerance of plants to abiotic stresses can be increased by overexpressing antioxidants and other components of the chloroplast protection network (see for example Badawi et al., 2004; Tang et al., 2006; Sun et al., 2010b; Sun et al., 2010a; Chang et al., 2016; Wang et al., 2018a; Wang et al., 2018b; Wang et al., 2020a; Wang et al., 2020b). However, it is clear that such protective systems are not designed to eliminate ROS but merely to prevent excessive and uncontrolled oxidation. Recent literature supports the concept that production of a relatively stable H_2_O_2_ pool in the chloroplasts might lead either to direct export of this oxidant or to the transmission of downstream redox signals from the chloroplasts. Multiple distinct pathways for ROS formation associated with the photosynthetic electron transport system have been demonstrated. Current data suggest that only the PSI acceptor side is able to generate sufficient H_2_O_2_ accumulation to contribute to retrograde signalling (Lima‐Melo et al., 2021). Studies using P700 spectroscopy combined with electron paramagnetic resonance spin trapping demonstrated that electron flow to PSI is essential for the accumulation of H_2_O_2_ (Fitzpatrick et al., 2021). H_2_O_2_ produced by chloroplasts transmits information to the nucleus in order to regulate gene expression (Leister, 2019).

The different ROS forms produced by the electron transport processes (^1^O_2_, H_2_O_2_ and O_2_^●−^) are characterised by their relative reactivities and lifetimes. As such they are likely to function as discrete redox signals that trigger different pathways to regulate the expression of specific suites of nuclear genes. O_2_^●−^ has a relatively low level of reactivity but it can react with ascorbate, plastocyanin and nitric oxide. Crucially, O_2_^●−^ radicals can inhibit the activities of FeS (4Fe–4S) cluster‐containing proteins by releasing iron. The chemical or enzymatic dismutation of O_2_^●−^ generates H_2_O_2_, which is removed by enzymes such as APX and PRX. However, these enzymes do not serve to completely eliminate H_2_O_2_ from the chloroplasts, but rather to prevent excessive accumulation of this oxidant. H_2_O_2_ signalling occurs primarily though the oxidation of protein Cys residues. The oxidation of the redox‐sensitive Cys thiols on proteins leads to the reversible formation of sulphenic acid (‐SOH) that is stabilised by forming a disulphide bond (S‐S) with a nearby thiol or a mixed disulphide bond with reduced GSH. ROS‐mediated Cys oxidation occurs on proteins that are located close to the sites of ROS production. ROS can react with diverse protein side chains and they have no specificity for reactive Cys. PTMs of Cys residues may occur either through dithiol–disulphide exchange reactions or through reactions in which particular protein Cys residues are oxidised by ROS, reactive nitrogen species or reactive sulphur species (Zaffagnini et al., 2019). It is likely that the reversible redox regulation of Cys residues on specific chloroplast proteins may facilitate precise and specific H_2_O_2_ signalling, as has been demonstrated for the plasmalemma‐localised HPCA1 protein (Laohavisit et al., 2020), as well as metabolic and functional regulation, but definitive evidence of such roles for chloroplast PRX and THX proteins is lacking. Reversible redox PTMs can act as a regulatory switch that can alter the interactome, enzyme activity, conformational integrity, signalling functions and protein stability in response to cellular redox state changes (Foyer, Kyndt, & Hancock, 2020). While H_2_O_2_ can pass from the stroma to the cytosol, recent evidence suggests that H_2_O_2_ can be directly transferred from chloroplasts to the nucleus through contact sites (Figure 5) under high light conditions (Exposito‐Rodriguez et al., 2017; Mullineaux et al., 2020). The accumulation of H_2_O_2_ can also induce stromule formation (Hanson & Hines, 2018), allowing direct transfer of H_2_O_2_ from chloroplasts to the nucleus (Figure 5; Caplan et al., 2015).

ROS are part of a network of retrograde signals that adjust chloroplast functions to prevailing metabolic and environmental conditions during organelle biogenesis and photosynthesis. Other chloroplast to nucleus signals that have been identified are triggered by accumulation of 3′‐phosphoadenosine 5′‐phosphate (Estavillo et al., 2011), methylerythritol cyclodiphosphate (Bjornson et al., 2017), dihydroxyacetone phosphate (Vogel et al., 2014) or heme (Espinas et al., 2012). 3′‐Phosphoadenosine 5′‐phosphate is a product of sulphur metabolism (Kopriva & Gigolashvili, 2016). Sulphotransferases transfer the sulphate group from phosphoadenosine phosphosulphate (PAPS) to other acceptor molecules such as desulphoglucosinolates and salicylic acid (Chan et al., 2013). The accumulation of 3′‐phosphoadenosine 5′‐phosphate is prevented by the activity of the SAL1/FRY1 phosphatase. This enzyme degrades 3′‐phosphoadenosine 5′‐phosphate to inorganic phosphate (Pi) and adenosine monophosphate (AMP) in chloroplasts and mitochondria. Oxidative inactivation of SAL1 leads to an accumulation of 3′‐phosphoadenosine 5′‐phosphate under stress conditions (Chan et al., 2016). Mitochondrion to nucleus and chloroplast to nucleus signalling are linked by this pathway through the regulation of ANAC013 and ANAC017 (Shapiguzov et al., 2019). These transcription factors mediate a ROS‐related retrograde signal originating from mitochondrial complex III in order to activate the expression of mitochondrial dysfunction stimulon (MDS) genes that include the alternative oxidases and *SOT12* (De Clercq et al., 2013). Several MDS genes including *SOT12* are also induced by the 3′‐phosphoadenosine 5′‐phosphate/SAL1 signalling pathway (Van Aken & Pogson, 2017). ANAC013 and ANAC017 functions are suppressed by the nuclear‐localised RADICAL‐INDUCED CELL DEATH1 (RCD1) protein. This nuclear hub protein acts as a co‐regulator of phytohormone and stress responses and links signalling from chloroplasts and mitochondria to control metabolism in both organelles (Shapiguzov et al., 2019).

Chloroplast antioxidants such as sAPX and thylakoid APX (tAPX) may play a role in the regulation of retrograde signalling that operates in plant responses to environmental stresses (Bittner et al., 2021), as well as the induction of nuclear‐encoded pathogen defence genes in the absence of any pathogen challenge (Maruta et al., 2012). tAPX is mainly localised in the unstacked regions of the thylakoid membrane, where it removes the H_2_O_2_ produced at PSI at the expense of ascorbate (Groden & Beck, 1979). Ascorbate is regenerated at the thylakoid membrane (Miyake & Asada, 1994) and also in the chloroplast stroma (Foyer & Halliwell, 1976). tAPX has higher turnover rates for H_2_O_2_ than PRX (Dietz et al., 2006). Ascorbate regeneration is coupled to the PETC by fd‐dependent monodehydroascorbate reduction (Miyake & Asada, 1994). tAPX protects PSII more efficiently from photooxidative damage than sAPX (Maruta et al., 2010). The role of the GSH pool and the involvement of dehydroascorbate reductases (DHARs) in regenerating ascorbate and detoxifying ROS has been called into question (Rahantaniaina et al., 2017). Nevertheless, a recent study has demonstrated that GSH is involved in recycling ascorbate under high light conditions either through non‐enzymic reduction or via DHAR activity (Terai et al., 2020).

The chloroplast TRXs and TRX‐like proteins also play a key role in this regulation, using the reducing power of the PETC to control ROS production at key electron transport components via ferredoxin/FTR and NADPH/NTRC, as well as regulating the activities of the redox‐sensitive stromal enzymes in response to light. The NTRC and FTR systems coordinate stromal metabolism and electron transport activity, as well as regulating ROS generation in response to changing environmental factors such as light intensity (Nikkanen & Rintamäki, 2019). This regulation also involves other redox components such as PRXs that control the reducing activity of chloroplast TRXs and facilitating rapid oxidation of stromal enzymes in the dark.

## REGULATION OF ^1^O_2_ PRODUCTION AND SIGNALLING

The generation of ^1^O_2_ by PSII is also regulated by the binding of bicarbonate (Brinkert et al., 2016), which has significant effects on PSII activity. Bicarbonate binds both to the electron donor side of PSII to regulate water oxidation and to the non‐heme iron, which is situated between the quinone cofactors called QA and QB that are located on the acceptor side of PSII. When electron acceptors are in short supply, the QA quinone becomes reduced, releasing bicarbonate. The loss of bicarbonate causes a positive shift in the redox potential of QA – decreasing the energy gap between QA and QB – and increasing the thermodynamic barrier for electron back‐transfer between QA and Pheo. The decreased rate of electron transfer decreases the likelihood of electron back‐transfer, which would result in chlorophyll triplet‐mediated ^1^O_2_ production (Brinkert et al., 2016). Some of the small carboxylic acids that are produced by the photorespiratory pathway in chloroplasts can replace bicarbonate and thus also modify the midpoint potential of QA (Messant et al., 2018). The reversible inactivation of PSII by glycollate provides a mechanism by which photorespiration can limit ^1^O_2_ production at PSII, in addition to acting as a safety valve for PSI (Shi et al., 2022).

Numerous studies have demonstrated that ^1^O_2_ is a powerful signalling molecule that regulates the expression of nuclear genes, leading to either programmed cell death or acclimation responses, depending on the intensity of the stress and the level of ^1^O_2_ production (Kim, 2020; Ramel et al., 2013). Two distinct spatially separated ^1^O_2_‐triggered chloroplast to nucleus signalling pathways have been characterised (Dogra & Kim, 2020). The ^1^O_2_ sensors in the thylakoid membranes are β‐carotene, which is localised in the PSII reaction centres within the core of the grana stacks (Ramel et al., 2013), and the EXECUTER1 (EX1) protein, which is localised within the non‐appressed grana margins (Wang et al., 2016). Within the grana stacks, ^1^O_2_ modifies gene expression through the oxidation of β‐carotene and the generation of carotenoid breakdown products such as β‐cyclocitral (β‐CC), β‐ionone or dihydroactinidiolide (dhA), some of which are volatile (Ramel, Birtic, Cuine, et al., 2012). β‐CC and dhA act as signalling molecules that elicit a genetic response leading to a marked increase in plant stress tolerance (Ramel, Birtic, Ginies, et al., 2012; Shumbe et al., 2014) (see Figures 2 and 3). A general feature of the transcriptomic response to β‐CC is an induction of genes related to cellular defence against stress and a downregulation of genes related to cell growth and development. The β‐CC pathway induces various detoxification mechanisms, including GSH *S*‐transferases (GSTs) and UDP‐glycosyltransferases (Ramel, Birtic, Ginies, et al., 2012). These participate in cellular xenobiotic detoxifying processes, which are also activated by the reactive carbonyl species that are generated by the spontaneous decomposition of lipid peroxides (Mano, 2012). β‐CC induces the SCARECROW LIKE 14 (SCL14)‐controlled xenobiotic detoxification pathway (D'Alessandro et al., 2019), in which SCL14 and TGAII‐type transcription factors modulate the expression of the chloroplast‐localised ANAC102, which in turn controls the expression of the ANAC002, ANAC031 and ANAC081 transcription factors that regulate the redox enzymes involved in the first phase of the detoxification response (D'Alessandro et al., 2019). Crosstalk between the β‐CC pathway and pathways triggered by the accumulation of phosphoadenosine phosphate has been proposed (D'Alessandro & Havaux, 2019). However, the β‐CC signalling pathway is independent of other chloroplast to nucleus retrograde signalling pathways such as the tetrapyrrole pathway and the EX1‐dependent pathway (Ramel et al., 2013; Shumbe et al., 2016). For example, the OXI1‐mediated pathway of programmed cell death in the *ch1* mutant is independent of the EX proteins (Shumbe et al., 2016). Moreover, the β‐CC‐induced and EX1–FtsH2‐dependent pathways share only a small number of genes (Dogra et al., 2017). However, the β‐CC signalling pathway may have several branches, at least one of which involves the small zinc finger proteins methylene blue sensitivity 1 (MBS1) and MBS2 (Shumbe et al., 2017).

The EX1 and EX2 proteins play an important role in the transmission of ^1^O_2_ signals (Wang et al., 2016). EX1 transmits signals from ^1^O_2_ produced in the margins of the grana stacks (Wang et al., 2016), where the repair apparatus for damaged/inactivated PSII reaction centres is localised. EX1 is associated with PSII reaction centre proteins, including D1 and D2, and FtsH2 proteases, together with protein elongation factors and chlorophyll biosynthesis enzymes (Wang et al., 2016). The pathway of ^1^O_2_‐induced retrograde signalling is suppressed by the stroma protein SAFEGUARD1 (SAFE1), which negatively regulates ^1^O_2_‐mediated stress responses at the grana margins (Wang et al., 2020a; Wang et al., 2020b). ^1^O_2_ induces SAFE1 degradation by a pathway involving chloroplast‐derived vesicles that protects the grana margins of the thylakoid membranes (Wang et al., 2020a; Wang et al., 2020b).

## FINAL REMARKS AND OPEN QUESTIONS

The question of whether oxygen is an essential electron acceptor for photosynthesis has been debated and the role of oxygen as an alternative electron sink in pseudocyclic electron flow is well documented. However, the importance of oxygen as an essential energy and electron acceptor for ROS production in chloroplasts has only been recognised more recently. The ROS produced by the PETC in the thylakoid membrane clearly play important roles in photodamage. However, they also contribute to oxidative signalling and environmental sensing, expanding chloroplast functions far beyond those associated with the regulation of photosynthesis.

Our current understanding of how ROS production at the different sites in the electron transport system is controlled remains limited. While multiple sites of ROS production, i.e. the PQ pool (Khorobrykh et al., 2015), PTOX (Heyno et al., 2009), Cyt *b*_6_ *f* complex (Baniulis et al., 2013) and PSI (Mehler, 1951), exist in the thylakoid membrane, the effects of environmental conditions on the relative production at each site remain uncertain. Accumulating evidence supports the concept of photosystem‐specific roles for ROS production in the ETI and PTI pathways of plant defence against pathogens. It appears that ROS generated by ETI are derived primarily from PSII, whereas PTI require PSI‐mediated electron transport and H_2_O_2_ production (de Torres Zabala et al., 2015). H_2_O_2_ produced by PSI thus underpins PTI whereas a rapid accumulation of ^1^O_2_ is associated with the elicitation of ETI. Further clarification of the mechanisms directing ROS signalling pathways and how signals originating at different complexes and in different compartments of the chloroplast are differentially transduced is important in understanding how plants deal with a variety of abiotic and biotic stresses.

All of the ROS generated by chloroplasts have the potential to act as signals that modulate gene expression as well as metabolism and protein functions through redox PTMs. However, ROS signalling pathways can only be triggered if appropriate ROS‐sensing proteins are present at or near the site of ROS production in order to transduce redox PTMs through appropriate signalling pathways. Within this context, the antioxidant systems in each cellular compartment serve to remove ROS that are in excess of the amount that are required for signalling or the regulation of metabolism, as their very chemical reactivity can cause unspecific and potentially damaging oxidation. Whether (and how) the oxidised and reduced forms of antioxidants such as ascorbate and tocopherols can themselves contribute to the transmission of signals is largely unexplored. There are many sulphhydryl‐containing proteins and peptides in chloroplasts that have the potential to act as H_2_O_2_ sensors and hence function in signal transduction, with GSH, peroxiredoxins and TRXs the most likely candidates (Liebthal et al., 2018; Zaffagnini et al., 2019). However, to date, no specific sulphhydryl‐containing proteins that function directly as H_2_O_2_ sensors in chloroplasts have been identified. As yet, relatively few redox modulated H_2_O_2_ sensors have been characterised in plants. The plasmalemma‐localised leucine‐rich repeat receptor‐like kinase HPCA1 (CARD1) was recently shown to be a sensor for extracellular H_2_O_2_ (Wu et al., 2020) and quinones (Laohavisit et al., 2020). Similarly, quiescin sulphhydryl oxidase homologue 1 (QSOX1) was found to act as a redox sensor that negatively regulates plant immunity (Chae et al., 2021). While it is an attractive concept that specific chloroplast H_2_O_2_ sensors transmit signals through redox PTMs or by translation of oxidative signals into other types of signals such as direct regulation of protein kinases, H_2_O_2_ may also be directly trafficked from chloroplasts to the cytosol and nucleus, as illustrated in Figure 5. Identifying chloroplast H_2_O_2_ sensors and how they transmit signals to the nucleus is a major hurdle that must be overcome in the next decade. Different redox PTMs determine the function and fate of many chloroplast proteins but slow progress in redox proteomics methodologies has until recently limited our understanding of how the different types of redox PTMs that operate in chloroplasts govern PET and signalling.

Information regarding ROS accumulation in cells experiencing stress is transmitted to distal tissues and organs through activated ROS production catalysed by the plasmalemma‐bound respiratory burst oxidase homologues (RBOHs). These signals are propagated over long distances throughout the plant in a rapid cell to cell communication process called the ‘ROS wave’ (Fichman et al., 2020, 2021; Zandalinas et al., 2020a,b). This cell to cell signalling system also involves membrane channels including aquaporins and Ca^2+^‐permeable channels belonging to the glutamate receptor‐like, mechanosensitive small conductance‐like and cyclic nucleotide‐gated families that amplify the systemic signal in each cell along the path of the systemic ROS wave. In addition, alteration of the plasmodesmatal (PD) pore size through regulation of PD‐localised protein 1 (PDLP1) and PDLP5 is required for the propagation of the systemic ROS signals (Fichman et al., 2020). Little is known about how chloroplast ROS production participates in such long‐distance signalling pathways but a growing body of evidence supports the hypothesis that they play an important role, particularly as environmental sensors (Yi et al., 2015; Guo et al., 2016; Wang et al., 2018a; Wang et al., 2018b; Zhou et al., 2019). Similarly, photosynthesis is not necessary for local ROS responses to high light conditions, which are, at least in part, mediated by RBOH activity (Xiong et al., 2021), and CO_2_ signalling pathways can modify immunity in ways that are largely independent of photosynthesis (Hu et al., 2021). The signalling network that incorporates ROS to transmit local and long‐distance signals is highly complex and requires much more intensive research in years to come, in order to target these processes for sustainable crop production under climate change.

Recent advances in high throughput thiol redox proteomics are starting to improve our understanding of redox mechanisms in chloroplasts. Of the thousands of potential TRX targets identified through *in vitro* chloroplast redox proteomic studies, only about 20 have been characterised in terms of redox regulation *in vivo* (Geigenberger et al., 2017; Zimmer et al., 2021). Most potential TRX targets are localised in chloroplasts, but some are located in the mitochondria and cytosol. Future progress in this area will rely heavily on advances in redox proteomic techniques and their integration with biochemical and molecular genetic approaches. Similarly, the development of new and improved chloroplast‐targeted genetically encoded biosensors that can be used together with site‐specific redox proteomics will provide an improved platform for identification and characterisation of reductive and oxidative signals produced by the PETC and its protein targets (Doron et al., 2021; Haber et al., 2021).

The following open questions concerning the mechanistic regulation of PETC functions and oxidative signal generation and transduction remain to be addressed. Filling in these gaps in current knowledge will be essential in order to develop new strategies for crop improvement by tailoring reductive and oxidative signal generation and perception to improve photosynthesis in crop plants under suboptimal environmental conditions.

1 Spatio‐temporal control of ROS production is achieved through specific regulation of key PETC components. In some cases, for example the NDH/PSC1 complex, the capacity of these components to contribute to the chloroplast ROS budget remains unknown.
2 The structural aspects of protein–protein interactions that dictate the specificity of O_2_^●−^, production and signalling remain to be elucidated. The various assembly states of the photosystems and the different PSII forms that are produced in the repair cycle are likely to vary in their capacities to generate ROS. Similarly, it remains to be determined whether thylakoid regulation involves the formation of biomolecular condensates, micron‐scale membraneless compartments formed by liquid–liquid phase separation and other interactions that constrain chemical reactions.
3 The demonstrated role of photorespiratory metabolites in the control of ^1^O_2_ production suggests the possibility that other metabolites may fulfil similar roles and that primary metabolism may exert feedback control on primary processes.
4 The regulation of fluxes through alternative electron transport pathways that modulate ROS production remains poorly understood and merits further characterisation. Similarly, the specific roles of the different TRX and PRX forms in this regulation remain to clarified.
5 The redox regulation of the PEP complex remains poorly understood, particularly regarding the functions of components such as TRXz, PAP4/FSD3 and PAP9/FSD2.
6 Redox control of chloroplast functions involves numerous intersecting pathways that may include specific ROS sensors, whose role is to transmit information concerning the energy status to the nucleus. Depending on environmental conditions, the relative activity of these pathways will vary in ways we are yet to fully understand.
7 The existence, nature and functions of ROS receptor proteins in chloroplasts remain to be demonstrated, as well as the mechanisms by which transmission of ROS signals from the chloroplasts to the nucleus and other cellular compartments is controlled.
8 Chloroplasts are rich in protein Cys residues that are able to undergo reversible oxidation. The functional operation of different redox PTMs such as *S*‐nitrosylation, *S*‐glutathionylation, *S*‐sulphhydration and *S*‐sulphenylation that can influence protein stability and localisation or instigate alternative protein–protein interactions and functions remains to be elucidated.
9 The redox state of the apoplast that is controlled by the levels of ascorbate and ascorbate oxidase (Karpinska et al., 2018) and by RBOH‐dependent H_2_O_2_ production (Guo et al., 2016) plays a key role in the acclimation of photosynthesis to changing irradiance, but little is known about how chloroplast ROS production influences systemic signalling through modulation of ROS waves and other signals.
10 Thiol‐based redox regulation plays a central role in determining photosynthesis‐related metabolic fluxes in dark/light transitions and responses to sunflecks and other transitions in irradiance. The relatively slow activation of CBC enzymes in response to such changes is a potential limitation to photosynthesis and hence crop yields. Is it therefore possible to modulate the activities of redox‐related components to ensure more effective activation and deactivation capacities?
